# Molecular Mechanisms of Phosphate Stress Activation of Pseudomonas aeruginosa Quorum Sensing Systems

**DOI:** 10.1128/mSphere.00119-20

**Published:** 2020-03-18

**Authors:** Xianfa Meng, Stephen Dela Ahator, Lian-Hui Zhang

**Affiliations:** a Guangdong Province Key Laboratory of Microbial Signals and Disease Control, Integrative Microbiology Research Center, South China Agricultural University, Guangzhou, China; b Guangdong Laboratory for Lingnan Modern Agriculture, Guangzhou, China; University of Iowa

**Keywords:** phosphate depletion, cell-cell communication, AHL, quorum quenching, PhoB

## Abstract

The hierarchical quorum sensing (QS) systems of Pseudomonas aeruginosa, consisting of *las*, *pqs*, and *rhl*, coordinate the expression of bacterial virulence genes. Previous studies showed that under phosphate deficiency conditions, two-component regulatory system PhoRB could activate various genes involved in cytotoxicity through modulation of QS systems, but the mechanism by which PhoR/PhoB influences QS remains largely unknown. Here, we provide evidence that among the key QS regulatory genes in P. aeruginosa, *rhlR*, *pqsA*, *mvfR*, and *lasI* were activated by the response regulator PhoB under phosphate-depleted conditions. We show that PhoB is a strong competitor against LasR and RsaL for binding to the promoter of *lasI* and induces significant expression of *lasI*, *rhlR*, and *mvfR.* However, expression of *lasI*, encoding the signal 3-oxo-C12-HSL, was increased only marginally under the same phosphate-depleted conditions. This seeming inconsistency was attributed to the induction of *pvdQ*, which encodes an enzyme for degradation of 3-oxo-C12-HSL signal molecules. Taken together, the results from this study demonstrate that through the two-component regulatory system PhoR/PhoB, phosphate depletion stress could influence the QS network by modulating several key regulators, including *lasI*, *rhlR*, *mvfR*, and *pvdQ*. The findings highlight not only the potency of the PhoR/PhoB-mediated bacterial stress response mechanism but also the plasticity of the P. aeruginosa QS systems in coping with the changed environmental conditions.

**IMPORTANCE** It is not fully understood how phosphate deficiency could influence the virulence of Pseudomonas aeruginosa through modulation of the bacterial QS systems. This report presents a systemic investigation on the impact of phosphate depletion on the hierarchy of quorum sensing systems of P. aeruginosa. The results showed that phosphate stress could have an extensive impact on the QS networks of this bacterial pathogen. Among the 7 QS regulatory genes representing the 3 sets of QS systems tested, 4 were significantly upregulated by phosphate depletion stress through the PhoR/PhoB two-component regulatory system, especially the upstream QS regulatory gene *lasI*. We also present evidence that the response regulator PhoB was a strong competitor against the *las* regulators LasR and RsaL for the *lasI* promoter, unveiling the mechanistic basis of the process by which phosphate stress could modulate the bacterial QS systems.

## Abstract (precis)

It is not fully understood how phosphate deficiency could influence the virulence of Pseudomonas aeruginosa through modulation of the bacterial QS systems. This report presents a systemic investigation on the impact of phosphate depletion on the hierarchy of quorum sensing systems of P. aeruginosa. The results showed that phosphate stress could have an extensive impact on the QS networks of this bacterial pathogen. Among the 7 QS regulatory genes representing the 3 sets of QS systems tested, 4 were significantly upregulated by phosphate depletion stress through the PhoR/PhoB two-component regulatory system, especially the upstream QS regulatory gene *lasI*. We also present evidence that the response regulator PhoB was a strong competitor against the *las* regulators LasR and RsaL for the *lasI* promoter, unveiling the mechanistic basis of the process by which phosphate stress could modulate the bacterial QS systems.

## INTRODUCTION

Pseudomonas aeruginosa is a ubiquitous opportunistic bacterial pathogen that can cause disease in plants and animals (1). In humans, P. aeruginosa takes advantage of immunosuppression and causes serious infections, especially in patients with cystic fibrosis and traumatic burns (2). Different sets of virulent factors are produced by P. aeruginosa during infection such as exotoxin A, elastase, rhamnolipids, phenazines, and effector proteins, which are collectively important for the establishment of bacterial infections. Research in the past 2 decades has revealed that most of these virulent factors are controlled by a sophisticated hierarchical quorum sensing (QS) system composed of regulators which respond to cognate signal molecules. In P. aeruginosa, three canonical and complete QS systems, i.e., *las*, *rhl*, and *pqs*, were identified 2 decades ago (3, 4). *las* and *rhl* encode the synthases LasI and RhlI, which produce *N*-3-oxo-dodecanoyl-homoserine lactone (3-oxo-C12-HSL) and *N*-butanoyl-HSL (C4-HSL), respectively, and their cognate transcriptional regulators LasR and RhlR (5). The third QS system, *pqs*, is composed of the signal 2-heptyl-3-hydroxy-4-quinolone (PQS) or its precursor, 2-heptyl-4-quinolone (HHQ), both of which bind to the transcriptional regulator MvfR (also known as PqsR). A set of genes, including *pqsABCD* and *phnAB*, are required for the biosynthesis of HHQ, which is converted to PQS with the participation of PqsH (6). In addition, a new intercellular communication signal was recently discovered by Lee et al. (7) and named IQS (integrated quorum sensing system), although its receptor has yet to be identified.

The *las* system is at the top of the QS hierarchy and controls the downstream QS systems and the virulence of P. aeruginosa in response to bacterial cell density. However, evidence is accumulating that LasR in P. aeruginosa strains isolated from human patients is frequently mutated (8, 9). Significantly, LasR mutation does not seem to forfeit *lasR*-controlled functions such as expression of RhlR and production of 3-oxo-C12-HSL and PQS (4, 9). Environmental stresses such as starvation and phosphate and iron depletion are known to promote the expression of QS genes and the production of virulence factors, allowing P. aeruginosa to adapt to either acute or chronic infection conditions (10–13). It has been shown that phosphate limitation could induce the production of IQS and consequently activate the *rhl* and *pqs* systems in the absence of a functional *las* system (7).

Phosphate is essential for all living cells as an essential component of the energy molecule ATP, nucleic acids, phospholipids in membranes, and other biomolecules. It is foreseeable that P. aeruginosa would face strong competition for free phosphate molecules and could respond to phosphate starvation conditions accordingly during infection. Substantial depletion of phosphate concentrations has been observed after surgical injury, which significantly increases the virulence of P. aeruginosa (14–16). A two-component system, comprising a histidine kinase sensor protein (PhoR) and a transcriptional response regulator (PhoB), controls the detection of and responses to phosphate stress (17, 18). Under phosphate limitation conditions, PhoB is phosphorylated on Asp^54^ (D^54^) by PhoR and thereafter activates the genes containing the PhoB binding site (the pho box) in their promoters.

Phosphate depletion was shown to affect the *rhl* and *pqs* QS systems through IQS signal and also the genes involved in the biosynthesis of rhamnolipids and phenazines (10, 19), but the mechanism of the influence of phosphate depletion on QS is not fully understood. In the present study, the expression level of QS regulatory genes under conditions of phosphate depletion and repletion was investigated, and the results unveiled extensive impact of phosphate stress on the QS networks of this bacterial pathogen. Among the 3 sets of QS systems tested, 4 regulatory genes, in particular, the upstream QS regulatory gene *lasI*, were significantly upregulated by phosphate depletion stress through the PhoR/PhoB two-component regulatory system. We also present evidence that the PhoB response regulator was a strong competitor against the *las* regulators LasR and RsaL for the *lasI* promoter. These results present a new insight into how phosphate depletion stress could modulate the bacterial QS systems.

## RESULTS

### QS regulatory genes are activated by phosphate depletion through PhoB.

**FIG 1:**
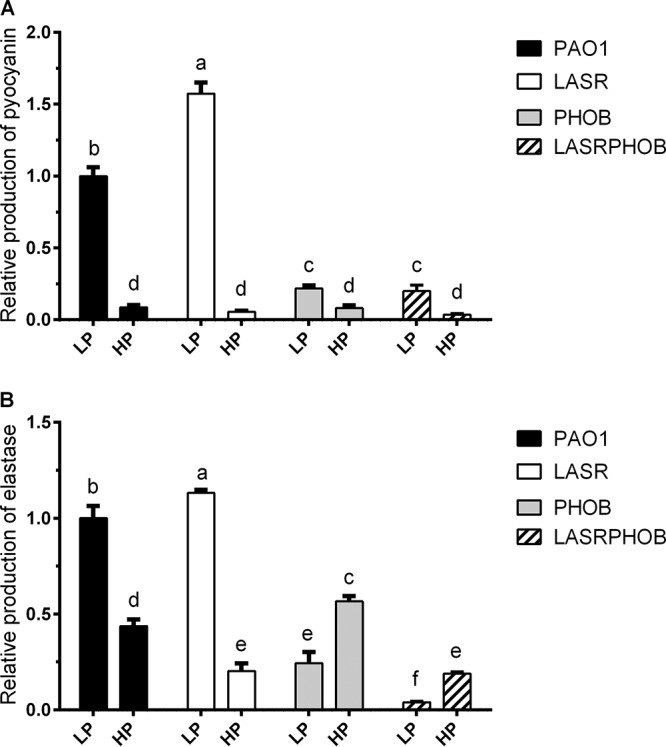
Relative production levels of elastase and pyocyanin in PAO1, LASR, PHOB, and LASRPHOB grown with LP and HP medium. The values of relative production represent averages of results from three biological replicates ± standard deviations. Distinct letters (a to e) indicate statistically different values calculated using two-way analysis of variance (ANOVA) (*P* < 0.05).

**FIG 2:**
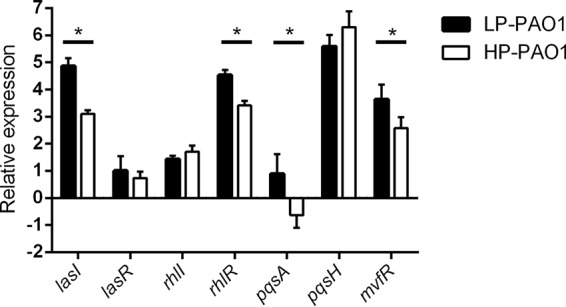
Relative expression levels of QS genes in wild-type strain PAO1 grown with LP and HP medium determined using RT-qPCR. The values of relative expression represent averages of results from three biological replicates ± standard deviations. Asterisks indicate a significant difference (two-way ANOVA, *P* < 0.05).

To understand the mechanisms by which phosphate depletion stress affects the bacterial QS system, production of pyocyanin and elastase was tested in P. aeruginosa wild-type strain PAO1, the *lasR* in-frame deletion mutant LASR, the *phoB* in-frame deletion mutant PHOB, and the *lasR*/*phoB* double in-frame deletion mutant LASRPHOB grown in low-phosphate (LP) and high-phosphate (HP) media (Fig. 1). In accordance with the results of a previous study (10), the production of pyocyanin was affected mainly by PhoB under phosphate-depleted conditions. As shown in Fig. 1B, production of elastase, which is controlled by the *las* and *rhl* systems (3), was also found to be influenced by PhoB. To evaluate the overall impact of phosphate depletion on P. aeruginosa QS systems, quantitative real-time PCR (RT-qPCR) analysis was carried out with the primers listed in Table S1 in the supplemental material. RNA samples were extracted from three biological replicates of PAO1 grown in LP and HP media. Of the seven genes tested, *rhlR*, *mvfR*, *lasI*, and *pqsA* were found to be significantly upregulated by phosphate depletion stress (Fig. 2).

10.1128/mSphere.00119-20.1 TABLE S1 Primers used in this study. Download Table S1, DOCX file, 0.02 MB. Copyright © 2020 Meng et al. 2020 Meng et al. This content is distributed under the terms of the Creative Commons Attribution 4.0 International license.

**FIG 3:**
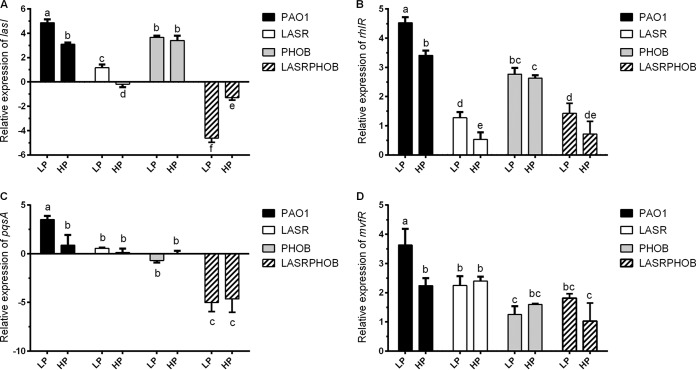
Relative expression levels of *lasI*, *rhlR*, *pqsA*, and *mvfR* in PAO1, LASR, PHOB, and LASRPHOB grown with LP and HP medium determined using RT-qPCR. The values represent averages of results from three biological replicates ± standard deviations. Distinct letters (a to e) indicate statistically different values calculated using two-way ANOVA (*P* < 0.05).

To determine how phosphate stress could influence QS, we compared the transcriptional levels of *lasI*, *rhlR*, *mvfR*, and *pqsA* by the use of PAO1, LASR, PHOB, and LASRPHOB, respectively. As shown in Fig. 3, the upregulation of the *lasI*, *rhlR*, *pqsA*, and *mvfR* genes under phosphate-depleted conditions was eliminated by deletion of the *phoB* gene, indicating that PhoB is important for the response to phosphate depletion. Expression of *lasI* and *rhlR* was statistically significantly activated by phosphate depletion in LASR, indicating that the regulatory effect of phosphate depletion on *lasI* and *rhlR* is independent of LasR. Furthermore, we found that expression of *lasI*, *rhlR*, *mvfR*, and *pqsA* presented at the lowest level in the *lasR*/*phoB* double deletion mutant LASRPHOB, especially under phosphate-depleted conditions (Fig. 3), which suggests that both LasR and PhoB are the key regulators of the bacterial QS systems.

**FIG 4:**
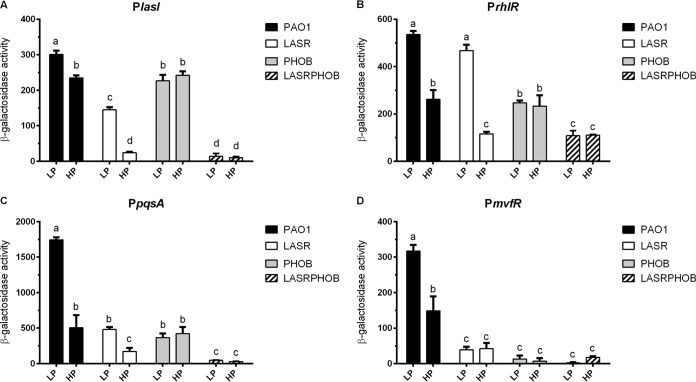
Promoter activities of *lasI*, *rhlR*, *pqsA*, and *mvfR* in PAO1, LASR, PHOB, and LASRPHOB grown with LP and HP medium. The values represent averages of results from three biological replicates ± standard deviations. Distinct letters (a to f) indicate statistically different values calculated using two-way ANOVA (*P* < 0.05).

To validate the RT-qPCR results described above, we examined the promoter activities of *lasI*, *rhlR*, *pqsA*, and *mvfR* in PAO1 and mutants LASR, PHOB, and LASRPHOB grown with LP and HP medium. The corresponding promoter fragments were amplified by PCR using the primers listed in Table S1 and were cloned into the gene fusion vector pME2-*lacZ* to control the transcriptional expression of the *lacZ* gene encoding β-galactosidase. In conformity with the RT-qPCR results, their promoter activities were significantly activated by phosphate depletion in PAO1 via PhoB (Fig. 4). Similarly to the results of Fig. 3, *lasI*, *rhlR*, and *pqsA* were significantly activated by phosphate limitation in the absence of *lasR*, confirming that the influence of phosphate depletion on downstream *rhl* and *pqs* QS systems was independent of the central *las* system. It was also noted that the deletion of either *lasR* or *phoB* exerted a much higher impact on *pqsA* and *mvfR* than on *lasI* and *rhlR* at the transcriptional level (Fig. 4). In agreement with the results shown in Fig. 3, double deletion of *lasR* and *phoB* significantly reduced the promoter activities of *lasI*, *rhlR*, *pqsA*, and *mvfR* under conditions of both phosphate depletion and phosphate repletion (Fig. 4).

### Effect of phosphate depletion on 3-oxo-C12-HSL production.

**FIG 5:**
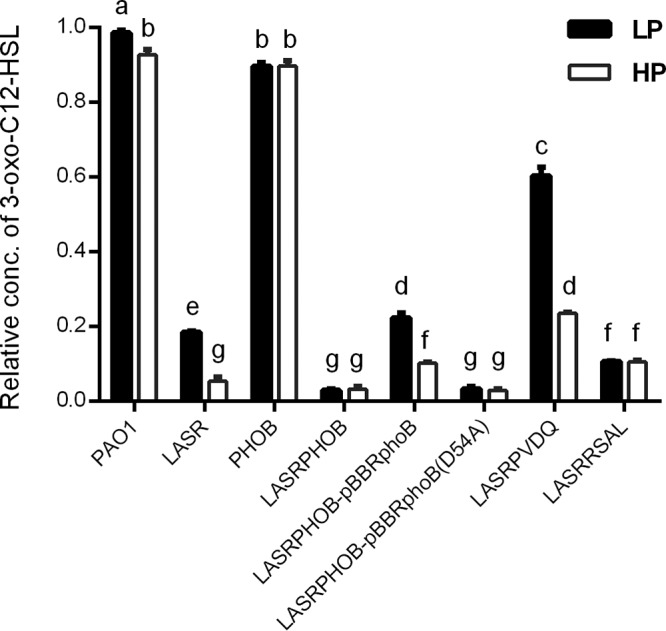
Relative concentrations of 3-oxo-C12-HSL in P. aeruginosa PAO1 and its derivate mutants grown with LP and HP medium. Concentrations of 3-oxo-C12-HSL from different samples were calculated against that from PAO1 grown with LP medium. The values represent averages of results from three biological replicates ± standard deviations. Distinct letters (a to g) indicate statistically different values calculated using two-way ANOVA (*P* < 0.05).

The RT-qPCR results and promoter activity analysis showed that *lasI* expression was activated by phosphate depletion through PhoB (Fig. 3; see also Fig. 4), which signifies a new regulatory pathway. The finding led us to test the regulatory effect of PhoB on the production of the *las* QS system signal 3-oxo-C12-HSL using wild-type strain PAO1 and its derivatives LASR, PHOB, and LASRPHOB. In addition, to determine whether phosphorelay signaling is associated with the regulatory effect of PhoB on *las* signal production, we engineered the construct pBBRphoB by placing the *phoB* coding region under the control of the pTac promoter in the expression vector pBBRMSC1-5. We generated the derivative pBBRphoB(D54A) from pBBRphoB by substituting residue D54 with alanine (A). The pBBRphoB construct and its derivative pBBRphoB(D54A) were transformed into the LASRPHOB double deletion mutant to generate the complemented strains LASRPHOB-pBBRphoB and LASRPHOB-pBBRphoB(D54A), respectively. Using the resulting strains, we analyzed the effect of phosphate depletion on 3-oxo-C12-HSL signal production (Fig. 5).

The results presented in Fig. 5 show that deletion of *lasR* led to a reduction in 3-oxo-C12-HSL production of about 95% under phosphate-replete (HP) conditions and a decrease of only 5% in the signal production in *phoB* mutant compared to the levels seen with wild-type strain PAO1. Under phosphate depletion (LP) condition, the deletion of *lasR* reduced the 3-oxo-C12-HSL level by about 80% whereas the deletion of *phoB* decreased its production by about 10% in comparison with the wild type. With reference to wild-type PAO1 and LASR, we observed an increase in 3-oxo-C12-HSL production under phosphate-depleted conditions compared to the replete condition (Fig. 5). The signal produced by LASR when grown in LP medium was 3.47 times higher than that in HP medium (Fig. 5). However, the deletion of *phoB* abolished the inducible effect of phosphate depletion as the double deletion mutant LASRPHOB was almost completely defective in the production of 3-oxo-C12-HSL under LP medium conditions. This indicates that the increased production of 3-oxo-C12-HSL in the *lasR* mutant under LP conditions compared to HP conditions was dependent on the presence of PhoB (Fig. 5). Consistent with the results described above, the effect of phosphate depletion on signal production in the LASRPHOB double deletion mutant was restored by the complementation of *phoB* and not by complementation of its allele *phoB*(D54A), suggesting that phosphorylation of Asp^54^ in PhoB is crucial for its biological function.

### PhoB competes against LasR and RsaL for binding with the *lasI* promoter.

**FIG 6:**
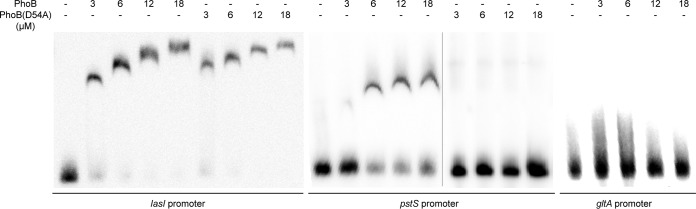
EMSA (electrophoretic mobility shift assay) analysis of phosphorylated and unphosphorylated PhoB on biotin-labeled *lasI* promoter. Various concentrations of PhoB (phosphorylated) and PhoB(D54A) (unphosphorylated) were applied. The *pstS* promoter and *gltA* promoter were used as the positive control and negative control, respectively. Images of *pstS* promoter were spliced for labeling purposes.

Since the expression of *lasI* had been proven to be regulated by PhoB, we further investigated the interaction between PhoB and the *lasI* promoter by electrophoretic mobility shift assay (EMSA) to determine its regulatory mechanism. The EMSA showed that PhoB bound to the promoter of *lasI* and that increasing the PhoB concentration seemed to reinforce its binding affinity (Fig. 6). The nonphosphorylatable PhoB(D54A), which lost its binding ability at the promoter of *pstS*, displayed a uniform binding pattern in a way similar to that seen with the unphosphorylated PhoB at the promoter of *lasI* (Fig. 6). The promoter of *pstS*, a key component in the phosphate-specific transport system (Pst), was shown previously by Hsieh and Wanner (20) to be the target promoter of the phosphorylated PhoB. These data indicate that phosphorylation of Asp(D54) in PhoB, which was demonstrated to be crucial for promoting *lasI* gene expression (Fig. 5), might not affect its binding ability to the *lasI* promoter.

**FIG 7:**
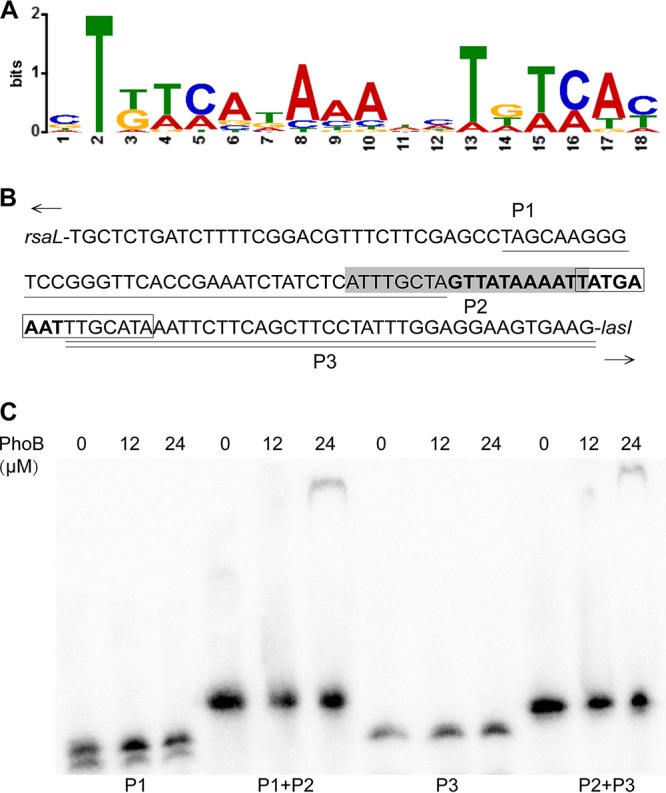
Validation of predicted binding site of PhoB at *lasI* promoter. (A) Consensus sequence of PhoB binding site derived from Escherichia coli *pho* box. (B) The *lasI* promoter, which contains binding sites for LasR (in shadow) and RsaL (in box), was artificially divided into three parts upstream of *pho* box P1 (with one line beneath) and *pho* box P2 (predicted *pho* box, in bold) and downstream of *pho* box P3 (with two lines beneath). (C) Electrophoretic mobility shift assay (EMSA) of different biotin-labeled probes in the presence of PhoB.

In order to identify the binding site of PhoB at the promoter region of *lasI*, we obtained the pho boxes of Escherichia coli from the PRODORIC database (http://prodoric.tu-bs.de/) (21). The PhoB binding motif was found with the MEME suite (http://meme-suite.org) (Fig. 7A). A survey for identification of a potential pho box at the *lasI* promoter was carried out by submitting the consensus sequence to MAST (http://meme-suite.org/tools/mast). An 18-bp sequence located in the *lasI* promoter was predicted to be the pho box (P2; Fig. 7B) and overlapped with the binding sites of LasR and RsaL. Among them, LasR is the cognate receptor of 3-oxo-C12-HSL and plays a positive regulatory role in transcriptional expression of *lasI*, whereas RsaL is a QS repressor which suppresses *lasI* expression by binding to its promoter (22). Validation of the predicted *pho* box was carried out by performing EMSAs with four DNA probes designed with sequences upstream of a *pho* box (P1), downstream of a *pho* box (P3), upstream of a *pho* box plus a *pho* box (P1+P2), and downstream of a *pho* box plus a *pho* box (P2+P3). The results in Fig. 7C showed that PhoB could bind to DNA fragments of P1+P2 and P2+P3 but not to P1 and P3, indicating that the predicted *pho* box (P2) is crucial for the binding of PhoB.

**FIG 8:**
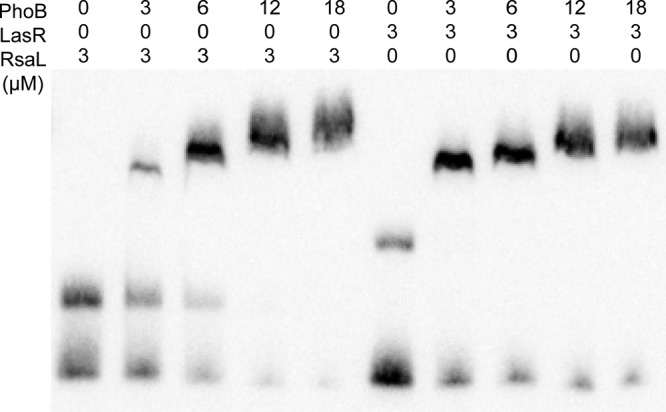
EMSA (electrophoretic mobility shift assay) analysis of biotin-labeled *lasI* promoter in the presence of diverse combinations of PhoB, RsaL, and LasR.

The potential interaction among PhoB, LasR, and RsaL in modulation of *lasI* was further investigated since their binding sites overlapped. A competitive binding assay showed that PhoB, at the same concentration of 3 μM, was able to compete effectively with RsaL for binding to the *lasI* promoter to form a unique band shift similar to that seen in the absence of RsaL and that further increasing the concentration of PhoB eliminated the RsaL-specific band shift (Fig. 8). In contrast, LasR was unable to bind to the *lasI* promoter in the presence of PhoB (Fig. 8). These results suggest that the binding affinity of PhoB is comparable with that of RsaL but is stronger than that of LasR with regard to the *lasI* promoter.

### Production of 3-oxo-C12-HSL under phosphate-depleted conditions was constrained by enhancing *pvdQ* expression.

**FIG 9:**
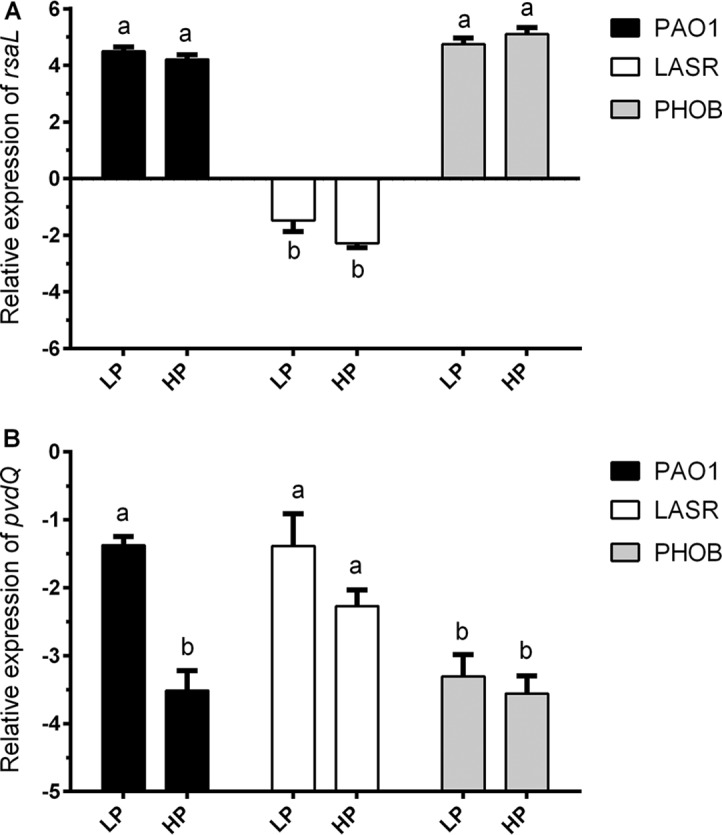
Activation effect of PhoB on *rsaL* and *pvdQ*. (A) Relative expression levels of *rsaL* in PAO1, LASR, and PHOB grown with LP and HP medium determined using RT-qPCR. (B) Relative expression levels of *pvdQ* in PAO1, LASR, and PHOB grown with LP and HP medium determined using RT-qPCR. The values represent averages of results from three biological replicates ± standard deviations. Distinct letters indicate statistically different values calculated using two-way ANOVA (*P* < 0.05).

The transcriptional level of *lasI* seen under phosphate-depleted conditions was four times higher than that seen under conditions of phosphate repletion (Fig. 2); however, this did not match with the increased ratio of 3-oxo-C12-HSL seen under the same conditions (Fig. 5). To resolve the puzzle, we investigated the regulatory effect of PhoB on RsaL, as RsaL was able to inhibit the transcription of *lasI* by dominating its promoter. Transcriptional analysis of *rsaL* reaffirmed that its expression was mainly controlled by LasR and that the effect of PhoB was negligible (Fig. 9A). If competition of RsaL against PhoB constrained the production of 3-oxo-C12-HSL, deletion of *rsaL* in LASR should result in higher signal production under phosphate-depleted condition. However, the *lasR*/*rsaL* double deletion mutant LASRRSAL exhibited low production of 3-oxo-C12-HSL and showed no significant differences under conditions of different phosphate levels (Fig. 5). These findings suggest that the inhibitory effect of RsaL was independent of PhoB and was not involved in constraining the production of 3-oxo-C12-HSL promoted by phosphate depletion.

We examined the effect of phosphate depletion on the expression of *pvdQ*, as PvdQ has been reported to be involved in 3-oxo-C12-HSL degradation (23). As is shown in Fig. 9B, the expression level of *pvdQ* in strain PAO1 grown with LP medium was over 4 times higher than that seen with HP medium. The effect of phosphate depletion on *pvdQ* transcriptional expression was abolished by the deletion of *phoB*, suggesting that phosphate depletion activated *pvdQ* via PhoB. The level of expression of *pvdQ* was significantly enhanced by deletion of *lasR* under phosphate-replete condition, which may imply a depressing effect of LasR. In agreement with the results of transcriptional analysis, the *lasR*/*pvdQ* double deletion mutant LASRPVDQ produced more 3-oxo-C12-HSL than LASR, especially under phosphate-depleted conditions, with levels reaching up to 60% of the signal produced by wild-type strain PAO1 (Fig. 5).

## DISCUSSION

As both QS and phosphate stress are involved in modulation of the virulence of P. aeruginosa, a systemic investigation was conducted in this study to investigate the impact of phosphate stress on QS systems. Among the 4 types of QS systems in P. aeruginosa, i.e., *las*, *rhl*, *pqs*, and *iqs*, the signal synthases and receptors or cognate regulators of *las*, *rhl*, and *pqs* have been identified and characterized (7, 24) and were thus included in this study. The results showed that phosphate stress could have an extensive impact on the QS networks of this bacterial pathogen. Among the 7 QS regulatory genes tested which are involved in the 3 canonical QS systems, 4 were significantly upregulated by phosphate depletion stress through the PhoR/PhoB two-component regulatory system. These 4 genes include *lasI*, encoding the *las* signal 3-oxo-C12-HSL; *rhlR*, encoding the receptor of the *rhl* signal C4-HSL; and *pqsA* and *mvfR*, whose products are involved in the biosynthesis of and response to the *pqs* signal PQS (5, 6). In addition, we have presented evidence that the two-component regulatory system PhoR/PhoB plays a key role in phosphate stress modulation of the P. aeruginosa QS systems. Our results unveiled the PhoB binding region in the promoter of *lasI* and demonstrated that the phosphate stress response regulator PhoB was a strong competitor against the *las* regulators LasR and RsaL for the *lasI* promoter. Moreover, we showed that phosphate depletion stress could also activate the expression of *pvdQ*, encoding an acyl-homoserine lactone (AHL)–acylase for degrading 3-oxo-C12-HSL signal, underlying the mechanism that explains why increased *lasI* expression did not translate into enhanced 3-oxo-C12-HSL signal accumulation under phosphate-depleted condition.

Evidence is accumulating indicating that phosphate depletion, which occurs following acute surgical injuries, could exacerbate virulence of P. aeruginosa (12, 14, 25). At least 10% of the genes in P. aeruginosa were found to be differentially regulated by phosphate starvation as shown by previous transcriptome analysis (15, 19), and many are known to be QS regulated (26, 27). Such an extensive impact of a phosphate regulon on gene expression and virulence is apparently associated with its ability to influence the bacterial QS networks. Significantly, our results showed that phosphate stress could affect multiple nodes of the QS networks in P. aeruginosa through the PhoR/PhoB two-component system. The hierarchical QS networks comprise the *las* system at the upstream position controlling the downstream *pqs* and *rhl* systems under normal *in vitro* conditions, while the *pqs* system plays an intermediary regulatory function in the *las-rhl* QS signal relay (3, 24), whereas under conditions of phosphate depletion stress, the *iqs* system can take over the role of the *las* system in regulation of the downstream *pqs* and *rhl* systems and the associated virulence factors (3, 7, 24). The results from this study, together with our previous findings on *iqs* (7), thus show that phosphate depletion stress could generate an extensive impact on the QS regulon by positively modulating the transcriptional expression of the key regulatory genes of all the 4 QS systems in the hierarchical QS networks of P. aeruginosa. Our data are consistent with previous transcriptional profiling assays showing that the phosphate regulon is involved in transcriptional activation of the *rhl* and *pqs* regulatory genes (19, 28) and that phosphate starvation can modulate P. aeruginosa virulence through influencing *rhl* and *pqs* QS circuits (10, 13, 15).

Transcriptional profiling and PhoB-based chromatin immunoprecipitation sequencing (Chip-Seq) analysis led to identification of 189 genes as representing the primary PhoB regulon in P. aeruginosa, with only three QS regulatory genes, i.e., *rhlR*, *pqsA*, and *mvfR*, found within this regulon (28), suggesting that PhoB could directly interact with their promoters and activate their expression under phosphate-depleted conditions. Another study using a bioinformatics approach identified 417 genes with a putative PhoB binding site in their promoter regions, including the QS regulatory genes *lasI*, *lasR*, *rhlR*, and *pqsR* (*mvfR*) of P. aeruginosa, and experiments confirmed that *rhlR* expression and PQS production were positively regulated by PhoB (10). Our results are highly consonant with the findings from the two studies cited above, except that we also found *lasI* to be part of the phosphate regulon (Fig. 2). We demonstrated that phosphate starvation stress could positively regulate the expression of *lasI* through the response regulator PhoB, which was confirmed by RT-qPCR and promoter activity analyses (Fig. 2, 3, and 4). Subsequent EMSA revealed that PhoB could bind to and compete with LasR and RsaL for the *lasI* promoter (Fig. 6, 7, and 8). This differs from the relationship of LasR and RsaL, which were able to bind to the promoter of *lasI* simultaneously without altering the binding affinity of one another and to activate or inhibit the transcription of *lasI* accordingly (22, 29, 30). Such different relationships of LasR versus RsaL and of PhoB versus LasR or RsaL are supported by the findings that the binding sites of LasR and RsaL on the *lasI* promoter are next to each other without apparent overlap whereas the binding site of PhoB covers part of that of LasR and RsaL, respectively (Fig. 7B). Our results also showed that PhoB had a binding affinity similar to that seen with the *lasI* promoter and RsaL but that PhoB displayed relatively stronger binding affinity than LasR (Fig. 8), which may suggest a dominant role of PhoR/PhoB over LasR in modulation of the QS regulon under phosphate-depleted conditions. Interestingly, unlike that at the *pstS* promoter, the binding affinity of PhoB at the *lasI* promoter did not seem to be altered by phosphorylation (Fig. 6), which is reminiscent of previously reported results of investigation of the response regulator VicR from Streptococcus mutans (31). Nevertheless, we showed that phosphorylation of PhoB was critical for its promotional activity upon transcriptional expression of *lasI* (Fig. 5). It is plausible that the phosphorylation affects its interaction with RNA polymerase in a way similar to that previously reported for the PhoP of Bacillus subtilis (32), a notion which awaits further investigations.

Curiously, the increased level of 3-oxo-C12-HSL accumulation seen under phosphate-depleted conditions (about 7%) (Fig. 5) did not seem to match the enhanced expression of *lasI* seen under the same conditions (over 50%) (Fig. 2). To explain the imbalance between *lasI* transcription and 3-oxo-C12-HSL accumulation seen under conditions of phosphate deficiency, we examined the expression patterns of *rsaL* and *pvdQ*, which encode the *lasI* repressor RsaL (22, 29, 30) and an AHL-acylase PvdQ degrading 3-oxo-C12-HSL (23, 33, 34), respectively. We found that only *pvdQ* expression was significantly induced by phosphate depletion stress (Fig. 9) and, consistently, that only deletion of *pvdQ* led to increased 3-oxo-C12-HSL production or accumulation (Fig. 5), suggesting that the promotional effect of PhoB on *pvdQ* neutralized its effect on increasing *lasI* transcription. It appears rational that P. aeruginosa might have evolved such flexible mechanisms which not only allow the bacterial cells to respond quickly to changes in the environment, but also enable them to maintain the 3-oxo-C12-HSL signal at a controllable threshold level to prevent overinduction or premature induction of downstream virulence genes.

In summary, this study conducted a systemic investigation of the impact of phosphate depletion stress on the transcriptional expression of QS regulatory genes in P. aeruginosa through RT-qPCR and promoter activity analysis. The results showed that phosphate depletion stress, via response regulator PhoB, is able to activate the QS networks of P. aeruginosa at multiple nodes, including the *las*, *rhl*, and *pqs* systems. These observations, together with previous findings revealing that PhoB could also impact *rhl* and *pqs* by stimulating the production of the newly discovered quorum sensing signaling IQS (7), suggest that phosphate regulon can modulate the expression of key regulatory genes of all 4 of the known QS systems in this bacterial pathogen. Among them, transcriptional expression of *lasI* was found to be directly controlled by PhoB as demonstrated by EMSA in this study and *pqsA* and *mvfR* were found to be most likely directly regulated by PhoB according to the results of Chip-Seq analysis (28), while *rhlR* was found to contain a putative PhoB binding site in their promoter regions (10). Such an extensive influence of the phosphate regulon on QS highlights not only the flexibility of the bacterial QS network in accommodating the environmental cues that benefit bacterial survival but also the complexity of bacterial QS systems and stress response mechanisms. In addition, these findings also emphasize the importance of investigating QS in various environmental settings. Apart from phosphate depletion, PhoB in P. aeruginosa was also found to be activated upon contact with differentiated human epithelial cells (35, 36), and QS is known to be affected by other environmental stresses, such as iron depletion (3). Understanding of these environmental cues and the underlying regulatory mechanisms may aid in developing effective therapies against bacterial infections. Phosphate supplementation has been proven to be effective in alleviation of a lethal phenotype caused by P. aeruginosa in intestinal and burn wound infections (37, 38).

## MATERIALS AND METHODS

### Bacterial strains and recombinant DNA techniques.

The bacterial strains used in this study are listed in Table S2 in the supplemental material. P. aeruginosa wild-type strain PAO1 and its derivatives were routinely grown at 37°C on Luria-Bertani (LB) medium with tetracycline 100 μg · ml^−1^ and gentamicin (Gm) 50 μg · ml^−1^ added if required. E. coli strains were grown at 37°C on LB broth, and, when required, appropriate antibiotics were added in the following concentrations: ampicillin 50 μg · ml^−1^; tetracycline 10 μg · ml^−1^; gentamicin 10 μg · ml^−1^; kanamycin 50 μg · ml^−1^. Low-phosphate (LP) media and high-phosphate (HP) media were described previously (7). All recombinant DNA techniques, including restriction digestion, agarose gel electrophoresis, purification of DNA fragments, and ligations with T4 DNA ligase, were performed as described previously (39). Triparental mating was conducted using the helper strain E. coli DH5α (pRK2013) with corresponding E. coli and P. aeruginosa strains.

10.1128/mSphere.00119-20.2 TABLE S2 Bacterial strains used in this study. Download Table S2, DOCX file, 0.02 MB. Copyright © 2020 Meng et al. 2020 Meng et al. This content is distributed under the terms of the Creative Commons Attribution 4.0 International license.

### Construction of P. aeruginosa derivatives.

The oligonucleotides and **p**lasmids used in this study are listed in Table S1 and Table S3, respectively. In-frame deletions of *phoB*, *rsaL*, and *pvdQ* were performed by allelic interchange with the use of a pK18GT suicide delivery system as previously described (40). Briefly, DNA fragments of the upstream and downstream flanking regions of target genes were amplified and cloned into pK18GT, respectively. The generated plasmids were transformed into E. coli DH5α and delivered to a P. aeruginosa *lasR* deletion mutant (LASR) by triparental mating. Allelic interchange colonies were selected on LB plates supplemented with 10% sucrose and verified by PCR analysis to confirm the generation of LASRPHOB, LASRRSAL, and LASRPVDQ, which were the deletion mutants of *phoB*, *rsaL* and *pvdQ* in LASR, respectively.

10.1128/mSphere.00119-20.3 TABLE S3 Plasmids used in this study. Download Table S3, DOCX file, 0.01 MB. Copyright © 2020 Meng et al. 2020 Meng et al. This content is distributed under the terms of the Creative Commons Attribution 4.0 International license.

Complementation of *phoB* in LASRPHOB was performed by using pBBRMSC1-5 (41, 42). *phoB* was cloned into pBBRMSC1-5, generating plasmid pBBRphoB, which was subsequently transferred to LASRPHOB by triparental mating, and the complementation strain LASRPHOB-pBBRphoB was selected in LB-Gm plates. Using overlap PCR, the codon of GAC in *phoB* was replaced by GCA, resulting in *phoB*(D54A), which encoded Ala^54^ instead of Asp^54^. Utilizing the same approach, a complementation strain called LASRPHOB-pBBRphoB(D54A) was generated in which phosphoacceptor Asp^54^ of PhoB was substituted by nonphosphorylatable Ala^54^.

### Relative production levels of pyocyanin and elastase.

The levels of production of pyocyanin and elastase were evaluated following a previously reported procedure (43). Briefly, P. aeruginosa strains were cultured in LP medium or HP medium overnight with shaking at 250 rpm at 37°C. To test the production of pyocyanin, 5 ml of culture supernatants was mixed with 3 ml chloroform and shaken vigorously for 30 min at room temperature. The solvent phase was mixed with 1 ml of 0.2 N HCl and shaken for an additional 30 min. The absorbance was measured at 520 nm and normalized against cell density (optical density at 600 nm [OD_600_]). To test the production of elastase, 500 μl bacterial-cell-free supernatants was mixed with an equal volume of elastin-Congo red buffer (100 mM Tris, 1 mM CaCl_2_, pH 7) containing 5μ · ml^−1^ elastin-Congo red (Sigma) and incubated for 2 h at 37°C with shaking at 200 rpm. The reaction mixture was centrifuged to remove insoluble elastin-Congo red, and the absorbance was measured at 520 nm and normalized against cell density at OD_600_.

### RT-qPCR to evaluate the effect of phosphate depletion on QS.

Total RNA samples were extracted from PAO1 and its mutants at the exponential phase grown with LP and HP medium (7). About 2 × 10^9^ cells were collected for RNA extraction using a Ribopure bacterial RNA isolation kit following the instructions of the manufacturer (Ambion Inc., USA). cDNA was generated with TransScript First-Strand cDNA Synthesis SuperMix following the protocol of the manufacturer (TransGen Biotech, China). The synthesized cDNA samples were quantified by the use of a NanoDrop 2000c system (Thermo Scientific), and the concentrations of the samples were adjusted to 10 ng·μl^−1^ with ultrapure water. Quantitative real-time PCR was performed by the use of a QuantStudio 6 Flex real-time PCR system (Thermo Fisher Scientific) using standard cycling mode. In each reaction mixture, 50 ng of cDNA template was mixed with PowerUp SYBR green master mix (Thermo Fisher Scientific) and with specific primers of target genes (Table S1) to reach a final volume of 15 μl. Each reaction was carried out for 2 min at 95°C for denaturation, followed by 40 cycles of amplification with 15 s at 95°C and 30 s at 60°C. Transcriptional data were normalized against the *rpoD* reference gene using the comparative celta cycle threshold (*C_T_*) method (44).

### Promoter activity analysis.

The promoter regions of *lasI*, *rhlR*, *pqsA*, and *mvfR* were amplified using primers with HindIII and EcoRI restriction enzyme sites (Table S1). The resulting DNA fragments were digested with HindIII and EcoRI and cloned in the same sites of vector pME2-*lacZ* (45). Electroporation was used to introduce the resultant constructs into strains PAO1, LASR, PHOB, and LASRPHOB. Transformants were then selected on LB agar plates containing tetracycline. Bacterial cells grown overnight at 37°C in LP and HP medium containing tetracycline were harvested for the measurement of β-galactosidase activities following a previously described method (46).

### Extraction and characterization of 3-oxo-C12-HSL.

Bacterial strains were grown with 100 ml LP and HP medium at 37°C under shaking conditions to an OD_600_ of about 1.0. Culture supernatants were collected by centrifugation and were extracted with an equal volume of ethyl acetate containing 0.1% (vol/vol) acetic acid. After removal of ethyl acetate from the organic phases by the use of a rotary evaporator (N-1100V-WB; Eyela, Japan), the residues were dissolved in methanol.

Quantification of 3-oxo-C12-HSL signals was carried out utilizing E. coli DH5α (pJN105L, pSC11) as described previously (47, 48). Briefly, the reporter strain was grown in LB broth containing ampicillin and gentamicin until an OD_600_ of 0.3 to 0.5 was reached. The cultures were then inoculated with l-arabinose to a final concentration of 0.04% along with the extracted samples. After a 2 h incubation, the concentration of 3-oxo-C12-HSL in each sample was determined by measuring β-galactosidase activities as previously described. Synthetic 3-oxo-C12-HSL was used as the standard.

### Protein expression and purification.

pET-28b (+) vector (Novagen) was used for expression of PhoB, RsaL, and LasR following the instructions in the pET system manual. Briefly, the coding sequences of PhoB, RsaL, and LasR without stop codons were amplified using the primer pairs listed in Table S1. The generated DNA fragments were cloned into NcoI-XhoI sites of pET-28b (+), thereby fusing each gene to the sequence encoding the C-terminal His tag. The resulting constructs, confirmed by sequencing, were subsequently transformed into E. coli BL21(DE3). E. coli BL21(DE3) cells carrying pET-PhoB or pET-RsaL or pET-LasR were grown in LB broth supplemented with kanamycin at 37°C until an OD_600_ of about 0.5 to 0.7 was reached. The temperature was then lowered to 18°C, and isopropyl β-d-thiogalactoside (IPTG) was added to reach a final concentration of 0.1 mM to induce the protein expression. For analysis of expression of LasR, 3-oxo-C12-HSL was required at a final concentration of 5 μM. After overnight incubation, cells were harvested by centrifugation and then frozen at –80°C. Bacterial pellets were thawed, resuspended in cold lysis buffer (50 mM NaH_2_PO_4_, 300 mM NaCl, 1 mM dithiothreitol [DTT], 10 mM imidazole, pH 7.8) containing protease inhibitors (Complete mini, EDTA free; Roche), and lysed by sonification. After high-speed centrifugation, the supernatant was incubated with 5 ml ProteinIso nickel-nitrilotriacetic acid (Ni-NTA) resin (TransGen Biotech, China) at 4°C for 1 h. The column was then washed with wash buffer (50 mM NaH_2_PO_4_, 300 mM NaCl, 1 mM DTT, 100 mM imidazole, pH 7.8) and eluted with the elution buffer (50 mM NaH_2_PO_4_, 300 mM NaCl, 1 mM DTT, 200 mM imidazole, pH 7.8). The protein purity was determined by SDS-PAGE analysis, and the pure protein samples were pooled and dialyzed against the analysis buffer (50 mM NaH_2_PO_4_, 300 mM NaCl, 1 mM DTT, pH 7.8) at 4°C. Using the same method, a mutein of PhoB in which the phosphoacceptor amino acid residue Asp^54^ was replaced by a nonphosphorylatable Ala^54^ residue was expressed and designated PhoB(D54A).

### Electrophoretic mobility shift assay (EMSA).

The PhoB binding motif was found with the MEME suite (http://meme-suite.org) using the data from the PRODORIC database (http://prodoric.tu-bs.de/) and was submitted to MAST to obtain a putative pho box at the promoter region of a *lasI* promoter. *pstS* promoters, *gltA* promoters, and *lasI* promoters of different lengths (listed in Table S4) were synthetized (Thermo Fisher Scientific). The DNA fragments were then labeled with biotin by using a biotin 3′-end DNA labeling kit following the instructions of the manufacturer (Thermo Fisher Scientific). A light shift chemiluminescent EMSA kit was employed for gel-shift experiments according to the instructions of the manufacturer (Thermo Fisher Scientific). The binding buffer contained 50 ng·μl^−1^ of poly(dIC), 10 nM biotin-labeled DNA fragment, and various concentrations of purified protein as indicated. Additional 3-oxo-C12-HSL was required to bring LasR to a final concentration of 2 μM immediately before applying EMSA. Binding reaction mixtures were incubated for 20 min at room temperature and separated by electrophoresis in 5% nondenaturing Tris-borate-EDTA (TBE) polyacrylamide gels in TBE buffer at 90 V. DNA was then electrophoretically transferred to a positively charged nylon membrane at 380 mA at 4°C for 30 min. After cross-linking was performed, the membrane with biotin-labeled DNA was blocked and detected by the use of a chemiluminescence readout, applying a Tano 5200 luminescent imaging workstation.

10.1128/mSphere.00119-20.4 TABLE S4 DNA fragments used for EMSA in this study. Download Table S4, DOCX file, 0.01 MB. Copyright © 2020 Meng et al. 2020 Meng et al. This content is distributed under the terms of the Creative Commons Attribution 4.0 International license.
