# Supplementary material for: Molecular Mechanisms of Phosphate Stress Activation of Pseudomonas aeruginosa Quorum Sensing Systems
Source: mSphere. 2020 Mar 18;5(2):e00119-20. doi: 10.1128/mSphere.00119-20 (PMC7082139; doi:10.1128/mSphere.00119-20)
Supplement: TABLE S1 [file mSphere.00119-20-st001.docx]

| Primers used for cloning purposes | | |
| --- | --- | --- |
| Plasmid | Target | Primer sequence (5’-3’) |
| pK-phoB | upstream flanking region | 5’-gctctagagccaggcttcttgtcctg  5’-ggcggccgggagcggggggtcttgcctcgggtcga |
|  | downstream flanking region | 5’-tcgacccgaggcaagaccccccgctcccggccgccc  5’-acgcgtcgaccgtcctgcggggtcttca |
| pK-rsaL | upstream flanking region | 5’-cgggatccctccagcgtacagtcggaa  5’-taagaagaacgtagcgcttgctctgatcttttcgga |
|  | downstream flanking region | 5’-tccgaaaagatcagagcaagcgctacgttcttctta  5’-ccaagcttccgaactggaaaagtggct |
| pK-pvdQ | upstream flanking region | 5’-cgggatccgtggagcgcgcgaccgccacc  5’-tctgataggcattgcttacgatgtcgtttctctgca |
|  | downstream flanking region | 5’-tgcagagaaacgacatcgtaagcaatgcctatcaga  5’-gctctagaagccgctgctcgttggccgcg |
| pBBRPhoB | *phoB* gene | 5’-cgggatccatggttggcaagacaatcct  5’-gctctagatcagctcttggtggagaaac |
| pBBRphoB(D54A) | *phoB*(D54A) the allele of *phoB* gene | 5’-cgggatccatggttggcaagacaatcct  5’-ggagcatccagagcaggatca |
|  |  | 5’-tgatcctgctctggatgctcc  5’-gctctagatcagctcttggtggagaaac |
| P*lasI* | promoter of *lasI* | 5’- tacaagcttcattgctctgatcttttcgga |
|  |  | 5’- tacgaattctcaacatgtaataaggactga |
| P*rhlR* | promoter of *rhlR* | 5’-cccaagcttggtgccgcaggtgctgctg |
|  |  | 5’-cggaattcgtggatcggctgcatctcgc |
| P*pqsA* | promoter of *pqsA* | 5’-cccaagctttcgcccagtgtactacgcaat |
|  |  | 5’-cggaattcgagaatgtaggtccggcatt |
| P*mvfR* | promoter of *mvfR* | 5’-cccaagcttgtgcgtcatagtcgctacacctgaag |
|  |  | 5’-cggaattcccgacggaccagctccacg |
| pET-RsaL | *rsaL* gene | 5’-taccatgggcatggcttcacacgagagaac  5’-ccctcgagctctctgatcttgcctctca |
| pET-LasR | *lasR* gene | 5’-taccatgggcatggccttggttgacggt  5’-ccctcgaggagagtaataagacccaaatt |
| pET-PhoB | *phoB* gene | 5’-taccatgggcatggttggcaagacaatc  5’-ccctcgaggctcttggtggagaaacgata |
| pET-PhoB(D54A) | *phoB*(D54A) the allele of *phoB* gene | 5’-taccatgggcatggttggcaagacaatc  5’-ggagcatccagagcaggatca |
|  |  | 5’-tgatcctgctctggatgctcc  5’-ccctcgaggctcttggtggagaaacgata |
| Primers used in the RT-qPCR experiments | | |
| Target gene | Primer sequence | |
| *ropD* | 5’-gcgaagaaggaaatggtc  5’-cgtaggtggagaacttgta | |
| *lasI* | 5’-cgtgctcaagtgttcaag  5’-gccagtggtatcgagaat | |
| *lasR* | 5’-tggatgctcaaggactac  5’-atggaagttcacattggc | |
| *rhlI* | 5’-tctctgaatcgctggaag  5’-ctcatggcgacgatgtag | |
| *rhlR* | 5’-catgatcgagttgctgac  5’-ttcttctggatgttcttgtg | |
| *pqsA* | 5’-aagacgatctgttcaaggt  5’-cagcaggatctggttgtc | |
| *pqsH* | 5’-gtagtgctgatcggtgac  5’-gattgcttcctgatgaactc | |
| *mvfR* | 5’-gttctgcgatacggtgag  5’-cgatggtgatggcgatat | |
| *rsaL* | 5’-ttcacacgagagaacacag  5’-atatagggaagggcaggtt | |
| *pvdQ* | 5’-agatgcacctgaccattc  5’-gaagtggctggaggtatc | |
