# Supplementary material for: Molecular Mechanisms of Phosphate Stress Activation of Pseudomonas aeruginosa Quorum Sensing Systems
Source: mSphere. 2020 Mar 18;5(2):e00119-20. doi: 10.1128/mSphere.00119-20 (PMC7082139; doi:10.1128/mSphere.00119-20)
Supplement: TABLE S2 [file mSphere.00119-20-st002.docx]

| Bacterial strains | | |
| --- | --- | --- |
| Strains | Relevant characteristics | Source |
| *E. coli* | | |
| DH5α | F^–^ Φ80*lacZ*ΔM15 Δ(*lacZYA^-^argF*) U169 *recA*1 *endA*1 *hsdR*17 (*r_K_*^–^, *m_K_*^+^) *phoA* *supE*44 λ^–^ thi-1 *gyrA*96 *relA*1 | TransGen Biotech |
| BL21(DE3) | F^-^ *ompT* *hsdS*(rBB^-^mB^-^) *gal* *dcm*(DE3) | TransGen Biotech |
| DH5α (pRK2013) | *E. coli* DH5α contains the helper plasmid pRK2013 | Laboratory collection |
| DH5α (pJN105L, pSC11) | The biosensor strain of 3-oxo-C12-HSL | Peter E. Greenberg |
| *P. aeruginosa* | | |
| PAO1 | Wild type strain | Laboratory collection |
| LASR | Deletion *lasR* mutant of PAO1 | Laboratory collection |
| PHOB | Deletion *phoB* mutant of PAO1 | Laboratory collection |
| LASRPHOB | Double deletion *lasR/phoB* mutant of PAO1 | This study |
| LASRRSAL | Double deletion *lasR/rsaL* mutant of PAO1 | This study |
| LASRPVDQ | Double deletion *lasR/pvdQ* mutant of PAO1 | This study |
| LASRPHOB-pBBRphoB | LASRPHOB complemented with pBBRphoB | This study |
| LASRPHOB-pBBRphoB(D54A) | LASRPHOB complemented with pBBRphoB(D54A) | This study |
