# Supplementary material for: Molecular Mechanisms of Phosphate Stress Activation of Pseudomonas aeruginosa Quorum Sensing Systems
Source: mSphere. 2020 Mar 18;5(2):e00119-20. doi: 10.1128/mSphere.00119-20 (PMC7082139; doi:10.1128/mSphere.00119-20)
Supplement: TABLE S3 [file mSphere.00119-20-st003.docx]

| Plasmids | | |
| --- | --- | --- |
| pK18GT | Broad-host-range suicide vector; *sacB*, Gm^R^ | Laboratory collection |
| pK-phoB | pK18GT containing the *phoB* flanking region with the gene being deleted in frame | This study |
| pK-rsaL | pK18GT containing the *rsaL* flanking region with the gene being deleted in frame | This study |
| pK-pvdQ | pK18GT containing the *pvdQ* flanking region with the gene being deleted in frame | This study |
| pBBR1MSC-5 | Broad-host-range cloning vector; Gm^R^ | Laboratory collection |
| pBBRphoB | pBBR1MSC-5 containing *phoB* | This study |
| pBBRphoB(D54A) | pBBR1MSC-5 containing *phoB*(D54A) the allele of *phoB* gene | This study |
| pME2-lacZ | Promoter activity detection vector; Tc^R^ | Laboratory collection |
| P*lasI* | pME2-lacZ containing promoter of *lasI* | This study |
| P*rhlR* | pME2-lacZ containing promoter of *rhlR* | This study |
| P*pqsA* | pME2-lacZ containing promoter of *pqsA* | This study |
| P*mvfR* | pME2-lacZ containing promoter of *mvfR* | This study |
| pET-28b(+) | His-tagged protein expression vector; Km^R^ | Laboratory collection |
| pET-RsaL | pET-28b(+) containing *rsaL* gene | This work |
| pET-LasR | pET-28b(+) containing *lasR* gene | This work |
| pET-PhoB | pET-28b(+) containing *phoB* gene | This work |
| pET-PhoB(D54A) | pET-28b(+) containing *phoB*(D54A) the allele of *phoB* gene | This work |
