# Supplementary material for: Molecular Mechanisms of Phosphate Stress Activation of Pseudomonas aeruginosa Quorum Sensing Systems
Source: mSphere. 2020 Mar 18;5(2):e00119-20. doi: 10.1128/mSphere.00119-20 (PMC7082139; doi:10.1128/mSphere.00119-20)
Supplement: TABLE S4 [file mSphere.00119-20-st004.docx]

| DNA fragments | |
| --- | --- |
| *lasI* promoter | TGCTCTGATCTTTTCGGACGTTTCTTCGAGCCTAGCAAGGGTCCGGGTTCACCGAAATCTATCTCATTTGCTAGTTATAAAATTATGAAATTTGCATAAATTCTTCAGCTTCCTATTTGGAGGAAGTGAAG |
| *pstS* promoter | TATCCCAAAACCCCTGGTCAGCGATGACCGGGGGTTTTGCTTTTGCGCGCGGAAAAAGGCGTGCTTCCTCGCTTCTTTTCTCGACTGCGAATGCCGGCGGCGACTGTCACAATTGCCCTGGAAACTACCCATCCTGCATGAAACGGATGGACGGCTACCTCCCCCAGGCGGATCGAAAACGCGCTCCAGAGGGCCTCCCGGGCCCCTCCGTGAAAGGCTGGAGCCCTTGTGTTTCCAGGCTTTTAGGGGTGTGGCAGGGCTTCTCCCAAGGCTTGCGGAAAAATGTCGGAAGGCTGATGGCATTTCATCCAACTGTCATATTCCTTTCATAGAGTCTTCATGCGGCCTGACGATACTGGCCCCCGTTCCATCCAACCCCATCCTGTCCAAGGAGCAAGGC |
| *gltA* promoter | ACTGAGAGCCCTAACGAACAGGGCTAGTTGTCGGGAAGTGCACTTGTCAGGTACCCATCACCAGGGAGTGGCGACCGTCTATGAAAGCGGGCCAGGAATACCCCTTGCGGTCGGACGCCGAGTATAGACAGTTAGGCTACTAATGACAACGAAAACACCCATCGCCAAATGGTTGATTGCGGGCCTTTTATAAAAGGCGTAAATAGCCCCCTTTTTCTGCGAAAACCCCGCCACGGAAGGCTCTACGGCATGGATTCGCGCAAATTGACTTTCGAATTTATCCCTCTATAGTGGTGCGGGCCCTGCGTGGGGGGTACTGATGATTTCAAGCATAAATAGGAGGCCATC |
| P1 | TAGCAAGGGTCCGGGTTCACCGAAATCTATCTCATTTGCTA |
| P1+P2 | TAGCAAGGGTCCGGGTTCACCGAAATCTATCTCATTTGCTAGTTATAAAATTATGAAAT |
| P3 | TTGCATAAATTCTTCAGCTTCCTATTTGGAGGAAGTGAAG |
| P2+P3 | GTTATAAAATTATGAAATTTGCATAAATTCTTCAGCTTCCTATTTGGAGGAAGTGAAG |
